# The Diagnosis of Tumours of the Central Nervous System

**Published:** 1946

**Authors:** Diana J. Kinloch Beck

**Affiliations:** Honorary Neurosurgeon, Royal Free Hospital, Elizabeth Garrett Anderson Hospital and Marie Curie Hospital, London; Regional Adviser in Neurosurgery to the South-West Region, Ministry of Health


### The Bristol Medico-Chirurgical Journal

*" Scire est nescire, nisi id me*
*Scire alius sciret."*

SUMMER, 1946.

### THE DIAGNOSIS OF TUMOURS OF THE CENTRAL NERVOUS SYSTEM

BY

Diana J. Kinloch Beck, M.B., B.S., F.R.C.S., F.R.C.S.E.

*Honorary Neurosurgeon, Royal Free Hospital, Elizabeth Garrett Anderson Hospital and Marie Curie Hospital, London ;*
*Regional Adviser in Neurosurgery to the South-West Region, Ministry of Health.*

This paper is concerned with the clinical diagnosis of tumours of the central nervous system : the pure clinical investigation which from long practice has come to include certain of the special methods, ophthalmoscopy, perimetry and lumbar puncture. Although the only true symptom of a tumour of the central nervous system is progressive loss of neurological function, gradual and of varying pace, it is well to remember that signs may be produced not only by invasion and indentation but also by focal or general anaemia, by abnormalities in the cerebro-spinal fluid circulation, and by shifts of the neuraxis with resultant pressure by bone, dural septa or stretched vessels.

The taking of the history is as important as it is difficult and time-consuming. It is a duty which pays high dividends, not only because the chronology of symptoms may provide the only clue to successful localization, but also because it captures the confidence of patients and relatives, so essential to success in treatment. Given a sound knowledge of medicine and pathology, the experience and patience necessary in the elicitation of useful information—and judgment in the acceptance or rejection of statements—it is possible in a large number of cases to make a provisional diagnosis on the history alone and, as it were, to direct future operations from that base.

B

The physical examination begins whilst we are interrogating the patient, in an assessment of his general condition, his mental attributes and his emotional stability, and in the observation of abnormalities of growth such as occur in craniopharyngeal tumours and in pituitary and pineal disorders. It proceeds to a thorough general examination designed to eliminate the presence of disease elsewhere likely to prejudice the outcome of a major operation or to be mistaken for a tumour. Amongst the latter are syphilis, arteriosclerosis, hypertension and renal disease, and, less commonly, hypoglycaemia, Alzheimer's disease and certain diseases of the blood. Examination of the skin may reveal abnormalities of texture and hair-distribution in pituitary and pineal disturbances, the *café-au-lait* patches or neurofibromata of von Recklinghausen's disease known to be associated with certain intracranial and spinal tumours, and the facial naevi found with certain vascular tumours of the brain. Since metastatic cerebral tumours are fairly common in the middle-aged, the rapid development of the symptoms of an infiltrating tumour or of multiple tumours of the brain (sometimes before the primary has given any sign) should cause a careful search for a primary or for the scar of an operation undertaken earlier for its removal.

There is no need for me to stress the importance of systematic examination of the central nervous system, beginning with all the cranial nerves and including the determination of visual acuity, the mapping out of the visual fields using test objects of several sizes, and the examination of the fundi for optic atrophy, papilloedema, or angiomata. In addition olfactory, auditory and vestibular tests and examination of the vocal cords should be carried out if the history suggests that they are indicated. Motor functions are investigated by noting muscular wasting or fibrillation, by observations on the posture, tone, power and co-ordination of the limbs and trunk, and by scrutinizing the position of the patient's head, his stance and his gait. Sensory functions are tested by cotton-wool, pin-prick, and appreciation of posture : abnormalities of any of these call for a more extensive examination. With the recording of the superficial and deep reflexes the neurological examination is at an end.

At this stage I usually examine the head, paying particular attention to its enlargement in the young, to areas of tenderness or of localized thinning or thickening of the bones. Dilatation of the veins of the upper lid is common in frontal tumours : localized enlargement of vessels on the shaven scalp may be of importance in diagnosis and in planning operation. Lastly, the skull should be auscultated for a bruit and its site of greatest intensity noted. Neurological examination should be repeated, for signs differ on successive days : following lumbar puncture their elicitation may be easier. In a confused or drowsy patient signs may only be unmasked after dehydration by rectal infusions or intravenous injections. Lumbar puncture on patients suffering from an intracranial tumour should be practised with discretion : in those exhibiting the symptoms or signs suggestive of a serious rise of intracranial pressure, it is absolutely contra-indicated, at least until the threshold of operation ; and even then the amount withdrawn should be no more than 2-3 c.cm. Its use in cases of suspected spinal cord tumour includes manometric observations. Pathological examination of the cerebro-spinal fluid is outside the scope of my subject.

### TUMOURS OF THE SPINAL CORD

Of the tumours affecting the spinal cord, 75 per cent. are benign : in the whole realm of surgery there are no better results than those obtained by their removal, provided the diagnosis is made soon enough. Those arising in the cord or its membrane are described as extra- or intradural, the latter group being subdivided into intramedullary and extramedullary. The onset of symptoms is usually gradual and dependent on the location of the tumour both as regards level and relationship to the cord, on the direction of growth of the tumour, and not a little on the mobility of the cord in movements of the spine and in forced expiratory activities.

The course of spinal-cord tumours is commonly chronic and progressive : in the life-history there are often but not invariably three phases. The first of these, the stage of involvement of nerve roots, is often absent. It is marked by pain which is characteristic and pathognomonic. It may precede other symptoms by months or years : it may be constant or intermittent, persist in a localized area or radiate. It is lancinating in character, aggravated by coughing, sneezing, lifting or straining, and invariably wakens the patient in the early hours of the morning : it is, indeed, often so severe as to compel him to walk the floor or to sleep sitting up. Many patients claim as their first symptom pain in a distant part of the body. It is, for example, not at all rare for cervical and thoracic cord growths to begin with pain in one or both legs, pain which is radicular neither in character nor in distribution. As a result of occipital, neck, shoulder, chest, back, abdominal or pelvic pain (the last three of which commonly persist for years before objective motor or sensory disturbances appear), these patients tend to fall into the hands of many physicians, including the psychologist and the gynaecologist.

Paraesthesiae, such as tingling, burning, numbness, pins and needles, and a sensation of cold have been insufficiently stressed as early symptoms. Their location is of considerable importance ; they usually begin first on the side opposite to that on which the tumour is subsequently found at operation. Subjective numbness occurs either as a root symptom, and is then on the same side, or as a result of cord compression, when it is on the side opposite to the tumour. It is perhaps pertinent to note that numbness spreading from the distal to the proximal part of a limb suggests the presence of an extramedullary tumour, whereas the reverse distribution indicates an intramedullary origin. A sensation of weight or heaviness in the abdomen or limbs is common, but I have the impression that girdle sensations are less frequent than we once supposed. It is only in a very small number of cases of cord tumour that subjective motor disturbances, such as weakness and stiffness, appear before the sensory ones.

The second stage is marked by beginning compression of the cord ; the signs naturally vary with the site of the tumour, and range, at least with extramedullary tumours, from the development of a Brown Séquard syndrome with antero-lateral tumours to ataxia consequent on interference with deep sensation with those situated posteriorly. It is necessary to emphasize that objective sensory disturbances are usually absent in the early stages, although there may be patchy areas of hyperaesthesia to pin-prick, heat and cold. Where several roots are involved, there is distinct diminution or loss of sensation. Later, the amount and extent of sensory disturbance depends on blocking or destruction of fibres in the cord and varies with the site of the tumour. In my experience, dissociation of sensation is more common in extramedullary than in intramedullary tumours, though where it occurs it is necessary to eliminate syringomyelia and haematomyelia as underlying causes. The demonstration of " sacral sparing " is as important in indicating compression of the cord from without as " saddle " anaesthesia is in pointing to a lesion of the cauda equina.

There are no more valuable signs than loss of joint-sense and of appreciation of vibration on the same side as the tumour. When the whole thickness of the cord is involved, these are always disturbed no matter where the tumour originated : the level of the tumour is then suggested by the level of the lowest spinous process at which vibration is felt. Localized tenderness of one or several spines below the tumour is also helpful.

In the early stages of the evolution of a cord tumour, loss of power with diminished tendon-jerks or lost cutaneous reflexes may have localizing significance. Muscle-fibrillation occurs fairly frequently both in wasted muscles and in those not so affected. Gradual compression of the pyramidal tracts results in well-known reflex changes, in increase of tone and diminution or loss of voluntary power below the level of the growth. In extramedullary tumours spasticity and weakness spread from the feet upwards : this never occurs in intramedullary growths, where the motor weakness spreads, as does the sensory disturbance, from centre to periphery.

Decubitus sores are common in cauda equina and conus tumours and in the third and final stage of cord compression, in which paralysis is usually complete, loss of sensation total, and the sphincters without control. The chances of cure by surgery have by now become remote.

I do not propose to discuss the clinical features of tumours at different levels : the diagnosis of the correct level depends on a careful history and examination followed by a critical survey of disturbed cord functions, be they motor, sensory or reflex.

### INTRACRANIAL TUMOURS

It is to be regretted that intracranial tumours are still regarded as rare and hopeless : as a result of this misconception many pass unnoticed, or their victims are denied treatment at a stage when it might well prove effective. Intracranial tumours are in fact amongst the commonest of all diseases of the central nervous system and constitute about 95 per cent. of intracranial space-occupying lesions.

All intracranial tumours are potentially urgent, no matter how mild their symptoms may be when they first come under observation. Grave symptoms may appear suddenly and are often unpredictable.

Before discussing diagnosis, I think it relevant to mention that some years ago Horrax[1] reported that his operative mortality had been reduced to 10-15 per cent. : that 56 per cent. of 400 consecutive tumours were completely and permanently removed, and 71 per cent. of the patients so relieved were leading useful lives with little loss of function.

Intracranial tumours cannot be discussed as a whole for two reasons : there is a great diversity of pathological type as regards histology and mode of growth, and there is a great variety in location. These two factors result in many different symptom-complexes. Broadly speaking, the intracranial tumours most easy to diagnose are the most malignant, and these are not worth touching surgically. The more favourable tumours are often difficult to diagnose, and to determine their presence, let alone their position, may tax all our resources.

It is important to realize that a patient with a brain-tumour may be extremely ill either because he has an unfavourable type of tumour or because a benign tumour has produced a great rise of intracranial pressure. It is clearly essential to decide which of these possibilities obtains in a given case. We therefore find ourselves seeking the answer to three questions : Is there a tumour ? Where is the tumour ? Of what type is the tumour ?

It is convenient to consider the diagnosis of intracranial tumours under the headings general and focal.

*General Signs :* Time was when headache, vomiting and papilloedema were regarded as the essential criteria of an intracranial tumour. They are in fact indicative of nothing more than a generalized rise of intracranial pressure : and although when a small tumour blocks the cerebro-spinal fluid circulation, for example at the foramina of Monro and in the third or fourth ventricles, they may be the first symptoms, it is dangerous ever to regard them as early.

An intracranial tumour should be suspected whenever without apparent cause a patient suffers repeated headaches, especially in the morning and increasing in frequency and severity. It should be suspected in children with vomiting increasing in severity and in adults with convulsions.

Drowsiness appears later and is never found in tumours apart from papilloedema, with the possible exception of those involving the hypothalamus. It is unusual for a slowing pulse and respiration and rising blood-pressure to occur with tumours except in the terminal phases.

The headache associated with an intracranial tumour is often characteristic. Bursting in type, it appears at first in the morning, is irregular and transitory, and gradually becomes a daily occurrence of some hours' duration. Later, it appears at other times and is increased by activities causing a rise of intracranial pressure. An expanding lesion within the skull should be suspected whenever there is recurring headache at even longer intervals.

The site of the headache is sometimes, but by no means always, of significance ; temporary relief may be afforded by vomiting which bears no constant relation to food, is often associated with severe nausea, and is commonest in the morning. Vomiting in children, whatever the cause, is apt to be embarrassingly unheralded, so we need not stress unduly its projectile character. Children may make little complaint of headache, perhaps because tumours in them so commonly occur in the posterior fossa and separation of the sutures compensates for a rising intracranial pressure.

Early in the course of papilloedema visual acuity is normal. In fact, the dissociation between the appearance of the discs and the acuity is striking. I underline this point because neglect of ophthalmoscopy is an obvious reason why tumours are overlooked until sight is grossly and often irrevocably damaged ; and also because it is useful in differentiating papilloedema from optic neuritis or retrobulbar neuritis, in which loss of vision is greater than the appearance of the discs would suggest. But absence of papilloedema does not necessarily mean that there is no tumour.

Generalized epilepsy is the first symptom in 18 per cent. of intracranial tumours : it differs in no way from the idiopathic variety and is specially common in slow-growing tumours. Its occurrence is of considerable value in differentiating cerebellar from cerebral lesions, for the seizures are quite different in type. The onset of generalized convulsive attacks after twenty is highly suggestive of an organic cause ; after thirty such cause is nearly always present, and tumour is far and away the most common.

*Focal Signs.* Little more than sixty years ago Rickman Godlee removed from the right central area a tumour localized on neurological manifestations alone. Since then it has become increasingly possible, in the words of Cushing, " not only to localize a lesion with considerable accuracy, but to foretell its histological character with reasonable certainty." In the last thirty years, we have seen the clear definition of the pituitary adenomata and the acoustic nerve tumour, the demonstration of visual field defects in temporal and occipital lobe tumours, the lucid description of the features of certain tumours of the cerebellum and of the syndromes associated with the meningiomata, whether occurring on the convexity of the brain or in any of the three cranial fossae.

In the time at my disposal I cannot do more than give an outline —and a sketchy one at that—of clinical localization. I shall begin for historical reasons with tumours of the central area. These are amongst the most easy to recognize because they produce Jacksonian seizures : a careful description of previous attacks and observation by the surgeon of the march of events is indispensible to the proper interpretation of the convulsive phenomena, which depend on the part of the pre-Rolandic gyrus most involved. Examination after a seizure is also required on account of the common occurrence of a post-epileptic paralysis, or Todd's paralysis. This may last for minutes or for hours, and the part most grossly affected affords us a useful clue. With extension of the tumour, there appear the contralateral defects in skilled movements and in the discriminative aspects of sensation, so familiar as to need no further description. Where, however, a tumour extends forward from the post-central area, some confusion may arise from finding paralysis of isolated movements with muscular atrophy and flaccidity in the presence of a positive Babinski if the leg area is involved.

Tumours of the frontal lobe are notoriously difficult to diagnose because the changes they produce can occur as the result of raised pressure from tumours situated elsewhere. If placed sufficiently far back, involvement of the motor, pre-motor and speech areas may break the silence ; but if to the involvement of the area concerned with conjugate eye-movements is added the tremor resulting from partial involvement of the caudate nucleus and basal ganglia, it may be wrongly concluded that the tumour lies in the posterior fossa. Because in anteriorly-placed tumours the signs of raised intracranial pressure are not outspoken, the idea of a tumour may not occur unless the patient has a fit or the doctor an ophthalmoscope. Affairs drag on until too late, that physiological milestone the climacteric bearing the blame for apathy, loss of fastidiousness, defects of memory, personality-changes, and disorders of conduct. Highly suggestive of a frontal lesion is incontinence of urine in a conscious patient. There are two neighbourhood signs of importance ; unilateral or bilateral anosmia (without a nasal cause) and the so-called Foster-Kennedy syndrome, in which there is optic atrophy on the side of the tumour and papilloedema on the opposite side.

Temporal lobe tumours are marked by visual hallucinations, uncinate fits often accompanied by smacking of the lips, upper quadrantic visual loss proceeding to complete hemianopia and weakness of the face on the opposite side. With medially-placed tumours paresis of the third nerve may occur, and when the dominant hemisphere is affected there is aphasia.

Occipital lobe tumours are uncommon and give rise to visual hallucinations and fits, often beginning with adversive movements of the head and eyes. There is found to be contralateral hemianopia in which macular vision is frequently spared. Extension forwards results in alexia, right-left agnosia, defective powers of calculation, spatial disorientation, and repudiation of the patient's own limbs on the opposite side.

A tumour of the pineal gland is almost certainly present when to the features of a rise of intracranial pressure are added loss of upward movement of the eyes, dilated inactive pupils, and at times absence of convergence. Occasionally there is third-nerve paralysis and gradually progressive loss of hearing.

The shift of emphasis from the endocrine changes to the combination of primary optic atrophy and hemianopia (usually bi-temporal) resulted in pituitary tumours being the first to be clearly defined clinically. These tumours must be diagnosed early not only to save sight, but also because extension beyond the sella results (according to Jefferson[2]) in an operative mortality of 33 per cent. Surgery holds no greater thrill than the recovery or restoration of vision and the filling out of the visual fields after removal of a pituitary tumour.

Mid-line and lateral-lobe tumours of the cerebellum present such distinct and well-known clinical features that I shall pass on to mention the tumours in the cerebello-pontine angle, of which the most common are the acoustic nerve tumours. The first symptom is unilateral deafness usually associated with tinnitus and often with vertigo ; many patients are unaware of the deafness until it is formally demonstrated, and so present themselves with the other two symptoms. The fifth cranial nerve becomes affected next with loss of corneal reflex and facial paraesthesia ; diplopia and weakness of the face follow, to be succeeded by dysarthria and dysphagia ; this first stage of cranial nerve involvement is followed by that of hydrocephalus and at a later stage by the symptoms of pressure on the pons and medulla.

I have of necessity drawn somewhat didactic pictures and am aware that in so doing I have told but part of the truth ; I hope, however, that I have shown that it is possible by accurate deduction to answer at least in part the questions I set myself earlier in this paper. There is a special exultation in occasionally being proved right by subsequent investigation and operative findings : and a stimulating challenge in the more common, if sobering, experience of being wrong.

REFEREENCES.

1 Horrax, G. (1943). *Bull. N.Y. Acad. Med.*, vol. 19., p. 125.
2 Jefferson, G. (1940). *P.R.S.M.*, vol. 33, p. 433.

